# An Experimental Investigation on the Kinetics of Integrated Methane Recovery and CO_2_ Sequestration by Injection of Flue Gas into Permafrost Methane Hydrate Reservoirs

**DOI:** 10.1038/s41598-019-52745-x

**Published:** 2019-11-07

**Authors:** Aliakbar Hassanpouryouzband, Jinhai Yang, Anthony Okwananke, Rod Burgass, Bahman Tohidi, Evgeny Chuvilin, Vladimir Istomin, Boris Bukhanov

**Affiliations:** 1 Hydrates, Flow Assurance & Phase Equilibria Research Group, Institute of Petroleum Engineering, School of Energy, Geoscience, Infrastructure and Society, Heriot-Watt University, Riccarton, Edinburgh EH14 4AS UK; 2 School of Geosciences, University of Edinburgh, Grant Institute, West Main Road, Edinburgh, EH9 3JW UK; 3 Skolkovo Institute of Science and Technology (Skoltech), 3 Nobel Street, Skolkovo Innovation Center, 10, Moscow, 143026 Russia

**Keywords:** Climate-change mitigation, Marine chemistry

## Abstract

Large hydrate reservoirs in the Arctic regions could provide great potentials for recovery of methane and geological storage of CO_2_. In this study, injection of flue gas into permafrost gas hydrates reservoirs has been studied in order to evaluate its use in energy recovery and CO_2_ sequestration based on the premise that it could significantly lower costs relative to other technologies available today. We have carried out a series of real-time scale experiments under realistic conditions at temperatures between 261.2 and 284.2 K and at optimum pressures defined in our previous work, in order to characterize the kinetics of the process and evaluate efficiency. Results show that the kinetics of methane release from methane hydrate and CO_2_ extracted from flue gas strongly depend on hydrate reservoir temperatures. The experiment at 261.2 K yielded a capture of 81.9% CO_2_ present in the injected flue gas, and an increase in the CH_4_ concentration in the gas phase up to 60.7 mol%, 93.3 mol%, and 98.2 mol% at optimum pressures, after depressurizing the system to dissociate CH_4_ hydrate and after depressurizing the system to CO_2_ hydrate dissociation point, respectively. This is significantly better than the maximum efficiency reported in the literature for both CO_2_ sequestration and methane recovery using flue gas injection, demonstrating the economic feasibility of direct injection flue gas into hydrate reservoirs in permafrost for methane recovery and geological capture and storage of CO_2_. Finally, the thermal stability of stored CO_2_ was investigated by heating the system and it is concluded that presence of N_2_ in the injection gas provides another safety factor for the stored CO_2_ in case of temperature change.

## Introduction

**Figure 1:**
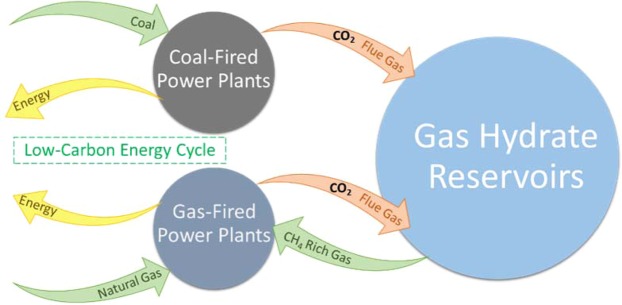
Graphical illustration of direct flue gas injection into hydrate-bearing sediments for geological carbon dioxide sequestration and methane recovery.

The oceans, permafrost regions, and continental and marine sediments contain a huge volume of methane trapped in the form of gas hydrates^1^ which could be a potential energy source^2^ or CO_2_ storage sink^3^, depending upon human actions, with respect to energy policy and anthropogenic global warming. These ice-like hydrates which are non-stoichiometric inclusion compounds with hydrogen bonded water cages enclathrated light guest molecules without chemical bonds^4^, require suitable thermodynamic conditions including pressure, temperature, and surrounding liquid and gas compositions to remain stable. Various approaches were taken to shift the system conditions away from equilibrium in investigations of methane extraction from hydrate-bearing sediments. In comparison with potential methods such as thermal stimulation^5^, depressurization^6^, chemical inhibitor injection^7^, CO_2_^8,9^ or CO_2_-mixed gases^10^ (e.g., flue gas) injection is more environmentally friendly because of the potential to capture CO_2_ simultaneously with methane recovery. Moreover, injection of CO_2_-mixed gases (mainly CO_2_ + N_2_) produced directly from power stations rather than pure CO_2_ is more economic owing to significant reductions in the total cost by avoidance of CO_2_ separation cost^10–12^ (See Fig. 1).

Coal-fired power plants represent a substantial proportion of global anthropogenic CO_2_ emissions which are the key contributors to global climate change^13^. The emission of CO_2_ in pulverized coal plants is caused by combustion of air and coal in a boiler to generate energy, producing flue gas with a low concentration of CO_2_ (~14%). Emission of flue gas from a typical 600 MWE power plant could be more than 500 m^3^ every second^14^. Accordingly, up-scaling post-combustion CO_2_ capture from power-plant flue gas through sustainable methods is gaining importance, to limiting CO_2_ emissions.

Previously, we presented initial results for injection of power-plant flue gas into gas hydrate reservoirs^15^ above freezing temperatures, defining a method^10^ to determine the optimum pressure for injection, where a more detailed review of the past literature can be found. In recent years, several^12,16–18^ investigations addressing various aspects of methane recovery by direct injection of flue gas have been reported, including limitations of CO_2_/CH_4_^16^, effects of methane hydrate morphology and ratio of CO_2_/N_2_ in the injection gas^17^, the effect of sandstone permeability^19^, and efficiency of the method in the association with thermal stimulation^12^. Here, referring to the companion papers^10,15^ as a basis for our interpretations where necessary, we report new experimental results for the kinetics of optimized flue gas injection into methane hydrate reservoirs at sub-zero temperatures, covering both sub glacial and permafrost conditions, in addition to further discussing previous results. The efficiency of the method at sub-zero temperatures was also investigated as a considerable proportion of gas hydrate reservoirs are located underneath permafrost formations^20–22^. In addition, our previous experiments above freezing point showed significantly more favourable results for both CO_2_ storage and methane recovery at lower temperatures, also emphasizing the necessity of investigating the method efficiency under 0 °C. One of the objectives of this study was to investigate the efficiency of the method (i.e., methane recovery and CO_2_ storage percentages) in a realistic time scale and at hydrate reservoir conditions. The second key objective was to evaluate the impact of reservoir conditions on the kinetic efficiency, to examine potential methane reservoirs and identify a suitable site. Finally, the impact of global warming and natural temperature cycles on the stored CO_2_ was also investigated with the aim of understanding potential environmental hazards.

## Results and Discussion

### Kinetics of CO_2_ capture and CH_4_ recovery at optimum pressure

**Figure 2:**
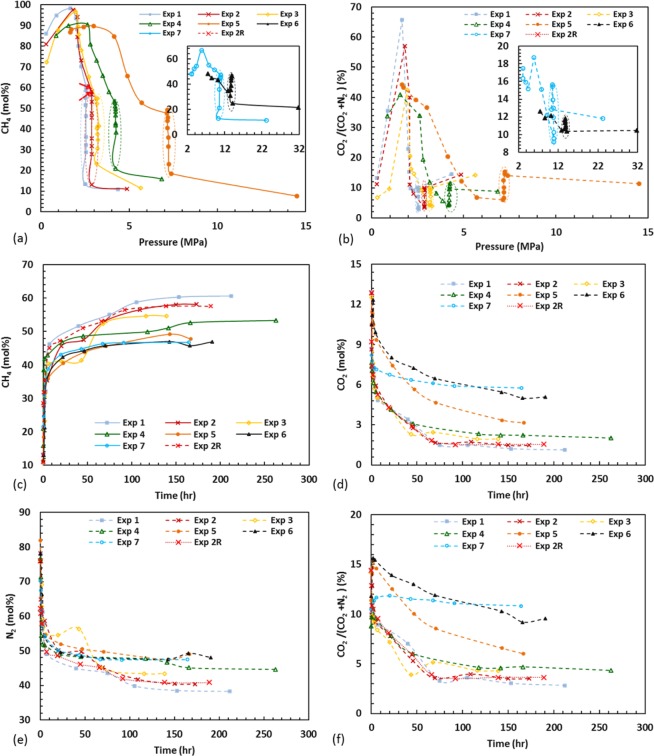
(**a**) CH_4_ concentration and (**b**) CO_2_/(CO_2_ + N_2_) evolution with pressure after flue gas was injected. CH_4_(**c**), CO_2_(**d**), N_2_(**e**), and CO_2_/(CO_2_ + N_2_) evolution with time after pressure was set to the optimum value.

Figure 2a–f show the changes in gas phase composition with time and pressure obtained by GC after flue gas was injected. As shown in Fig. 2a,b methane concentration and CO_2_/(CO_2_ + N_2_) ratio changes with pressure in all experiments following a similar pattern. However there were differences in the rate of change, especially for those experiments conducted at temperatures below 0 °C. Initially, these values fluctuate slightly as a consequence of the initial pressure reduction, made in order to set the system at target pressure. Methane concentration in the gas phase increases continuously and the CO_2_/(CO_2_ + N_2_) reduces at the target pressure until the system reaches equilibrium, as is indicated in circles in Fig. 2a,b. Gas concentration changes with time since the pressure set was plotted at Fig. 2c–f. The main mechanism involved is the chemical potential shift of the system to higher pressure after flue gas injection (see EXPERIMENTAL SECTION), which forces the methane molecules vacate the clathrate cages allowing CO_2_ molecules to enter. This could either occur through CO_2_ replacement or full/part dissociation of the existing methane hydrates and formation of new CO_2_ or CO_2_-mixed hydrates with an accompanying release of hydrogen-bonded water molecules. Considering the experimental conditions, formation of CO_2_, CO_2_-N_2_, CO_2_-CH_4_, CO_2_-N_2_-CH_4_, and N_2_-CH_4_ is possible from a thermodynamic point of view as the pressure in all the experiments is well inside the aforementioned hydrate stability zones (see EXPERIMENTAL SECTION).

Here, we define half-time (t_1/2_) as the time required for half of the increase in methane concentration to occur between when the pressure is set and the final equilibrium point. The defined half-time, t_1/2_ qualitatively reflects the economic viability of the proposed process for certain reservoirs, as for a reservoir with too high (t_1/2_) industrial scale application of the method would not be possible. t_1/2_ for all of the experiments was calculated to be no longer than 3 hours whereas the overall process could take up to 250 hours, indicating higher driving force due to higher chemical potential difference between gas and hydrate phase at the initial stage. A similar trend for the concentration of CO_2_ and N_2_ in the gaseous phase was observed (see Fig. 2d). At the later stage, increase of methane concentration in the gaseous phase dilutes CO_2_ and N_2_ concentration and consequently reduces the driving force for removing the CH_4_ molecules from the hydrate phase. It is also possible that replacement occurs rapidly on the surface of the methane hydrate, then the replacement rate decelerates considerably because of formation of a CO_2_-CH_4_ or CO_2_-N_2_ hydrate (CNH) layer on the surface, acting as a physical barrier. This layer prevents the CO_2_ molecules from entering into the interior of the methane hydrate, slowing down the replacement process. Thus, after forming a layer of CO_2_-CH_4_ or CNH, replacement slows down and a limited diffusion transport becomes the main mechanism for the replacement process. A similar explanation was presented in the literature^23–25^.

It is known that during formation of CO_2_-N_2_ mixed hydrate, CO_2_ goes to the larges cavities, whereas N_2_ will fill the small cavities^26^. To examine the selectivity of the CO_2_ over N_2_, CO_2_/(CO_2_ + N_2_) ratio with time after pressure set is presented in Fig. 2f showing a decrease with time, indicating stronger selectivity of the CO_2_ over N_2_ in all the experiments owing to relatively higher^27,28^ stability of CO_2_ than N_2_ at the hydrate phase. This shows relatively higher occupancy of large cavities than small cavities. These results are in agreement with previous studies^15,29^.

### Methane recovery during depressurisation

As mentioned in the previous section the system was depressurized stepwise every 24 hours after reaching equilibrium state at the target pressure. As shown in Fig. 2a, as the system pressure reduces, CH_4_ comes out of the hydrate phase and the percentage of CH_4_ in the gas phase increases until reaching the CO_2_ HSZ, after which CH_4_ started to decrease because of dissociation of the CO_2_ rich clathrates. This is true apart from Experiment 7, where CH_4_ in the gas phase increases after initial drop. This can be attributed to the fact that in this experiment the pressure of the CO_2_ hydrate dissociation is higher than that of CH_4_ hydrate (see EXPERIMENTAL SECTION), thus, CO_2_-rich hydrates dissociate first, followed by dissociation of CH4-rich hydrate. Regarding the CO_2_/(CO_2_ + N_2_) ratio during step-wise depressurization, Fig. 2b shows upward trends until passing CO_2_ HSZ. This could be justified by the fact that, during depressurization N_2_-rich gas was removed, and consequently overall CO_2_/(CO_2_ + N_2_) ratio was increased as a consequence of dissociation of those hydrates with more CO_2_ than N_2_, and after passing CO_2_ HSZ this ratio reached to its maximum amount because of the dissociation of CO_2_-rich hydrate. The maximum peak of this graph could be used as an indicator of efficiency of the CO_2_ capture at the optimum pressure, in that more CO_2_ stored at the optimum pressure means more CO_2_ trapped in hydrate phase so that this peak will be higher because of dissociation of more CO_2_ hydrate.

### Effect of hydrate reservoir temperature on CO_2_ sequestration

**Figure 3:**
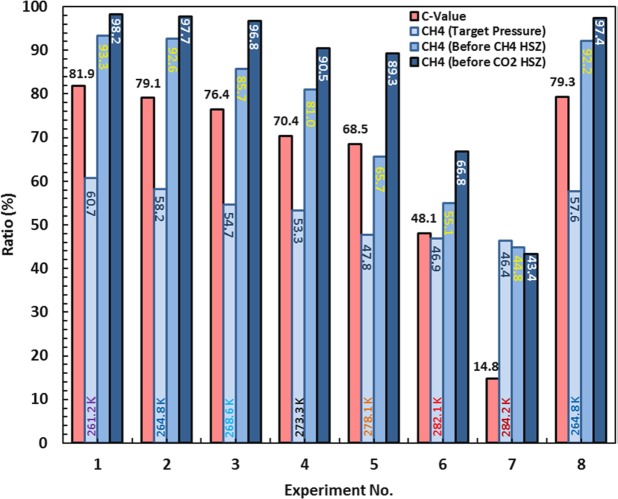
Calculated C-value, and CH_4_ concentration at the optimum pressures after the system reached to the equilibrium and CH_4_ concentration just before the system passed outside the CH_4_ HSZ and CO_2_ HSZ.

Both methane recovery and CO_2_ storage strongly depend on hydrate reservoir temperature. The extent of changes in gas compositions at lower temperatures are typically larger than those at higher temperatures, as would be expected due to the stronger selectivity of CO_2_ to N_2_ and CH_4_ in hydrate phase at lower temperature that dominates the molecular exchange between the gas and hydrate phase. In addition, the experimental pressures for lower temperatures were also lower than those for higher temperatures (see EXPERIMENTAL SECTION) due to the fact that hydrate dissociation points have lower pressures at lower temperatures. As a result, the compression cost for the reservoir with lower temperatures could be considerably lower. Although, lowering the temperature increases the time required for the process after (t_1/2_), graphs for both methane recovery and CO_2_ storage at negative temperatures show better efficiency in terms of time than positive ones at any times. With this in mind, the experiment at 261.2 K has the maximum efficiency for both methane recovery and CO_2_ storage, indicating that the more CO_2_ storage the more methane production. To be able to quantitatively analyse the results, C-value, is defined and referred to as the molar ratio of stored CO_2_ in the hydrate phase after reaching equilibrium at target pressure to the injected total CO_2_. C-value is calculated for each experiment using our in house thermodynamic modelling software^30^ and is plotted in Fig. 3 together with CH_4_ concentration in gas phase at target pressure after reaching equilibrium, before passing CH_4_ HSZ, and before passing CO_2_ HSZ. As can be seen 81.9% of the injected CO_2_ present in the flue gas was stored in the hydrate phase at −12 °C, and the molar percentage of CH_4_ in the gas phase reached 60.65, 93.34, 98.18 at the optimum pressure, and the boundary of CH_4_ HSZ and CO_2_ HSZ, respectively. For the experiments at higher temperatures, however, the figures in Fig. 3 decreased and reached a minimum in Experiment 7.

### Response of CO_2_-mixed hydrates to temperature rise

**Figure 4:**
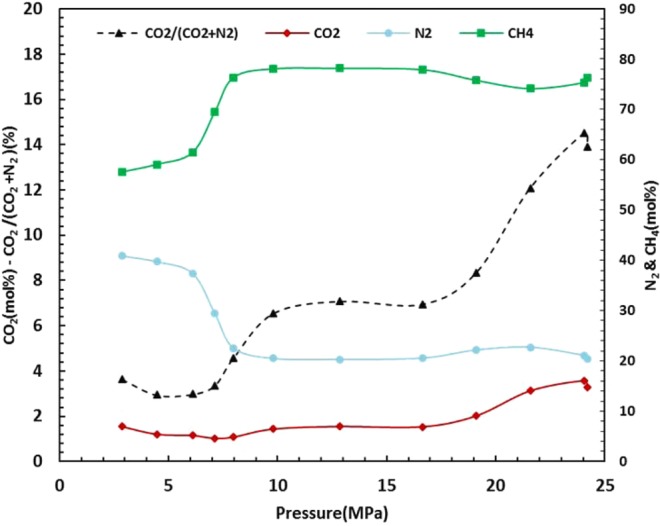
Gas compositional changes with pressure after cryostat temperature was set to 294.15 K at Experiment 2 R.

Thermal stability of the stored CO_2_ is also a major issue for the long-term stability of the CO_2_ underground because the temperature cycle in the storage environment may change the gas hydrates. This was the main reason for the investigation into the dissociation of hydrates in Experiment 2 R using thermal stimulation in addition to investigating the repeatability of the procedure. As can clearly be seen in Fig. 2, before depressurization Experiments 2 and 2 R showed very repeatable results. As mentioned before for Experiment 2 R, the temperature of the system was increased to investigate the dissociation behaviour of the previously formed hydrates at the optimum pressure. The gas composition evolution during dissociation was analysed by GC and the compositional results are plotted versus pressure in Fig. 4. As a result of temperature rise the gas phase starts to expand and the formed hydrates start to dissociate, which in turn leads to changes in the composition of the gas phase. As shown, initially the concentration of N_2_ increases whereas the concentrations of CH_4_, and CO_2_ and CO_2_/(CO_2_ + N_2_) reduces. The reduction of CO_2_/(CO_2_ + N_2_) shows that initially those hydrates with relatively more N_2_ start to decompose. This is a positive sign as it shows N_2_ not only acts as promoting agent for CO_2_/CH_4_ replacement but also provides another safety factor for retention of the CO_2_-rich hydrates during temperature rise. We have previously showed^31^ the role of N_2_ for providing safety factor for thermal stability of stored CO_2_ by hydrate formation using flue gas in absence of initial methane hydrate in place. However, this is the first mention of the potential safety role of N_2_ for methane recovery by flue gas injection. After this phase, there is a sharp change in the composition of the gas phase before reaching an almost stable composition. In this period, the gas hydrates are quickly dissociating and the released gas is going to the gas phase, thus, the absence of changes in the gas phase implies that the composition of the gas phase in this period is similar to the composition of those hydrates which are dissociating. After this phase, there is another sharp increase followed by a slight decrease in CO_2_ concentration and CO_2_/(CO_2_ + N_2_) ratio before the system reaches equilibrium. This rise shows that hydrates with higher CO_2_ content dissociate after dissociation of hydrates with lower CO_2_ content. The possible explanation for the slight decrease in the concentration of the CO_2_ and CO_2_/(CO_2_ + N_2_) ratio is the higher solubility of CO_2_^32^ compared to N_2_ and CH_4_.

## Experimental Section

### Materials

Deionized water (total organic carbon < 5 ppb) from an ELGA DV 25 Integral Water Purification was used for wetting sands, and cleaning of the experimental setup. The following gases from BOC Limited were used in the experiments: CO_2_ (99.995 vol %), N_2_ (99.995 vol %), and CH4 (99.995 vol %). Well-characterized sands from Fife, Scotland were used for simulating mesoporous hydrate reservoirs. As described in our previous work^31^, the Sand mainly consists of quartz which has very small gas adsorption capacity^33^ compared to gas inclusion in clathrate hydrate and water. Accordingly, the effect of gas adsorption to the sand was neglected in this study.

**Figure 5:**
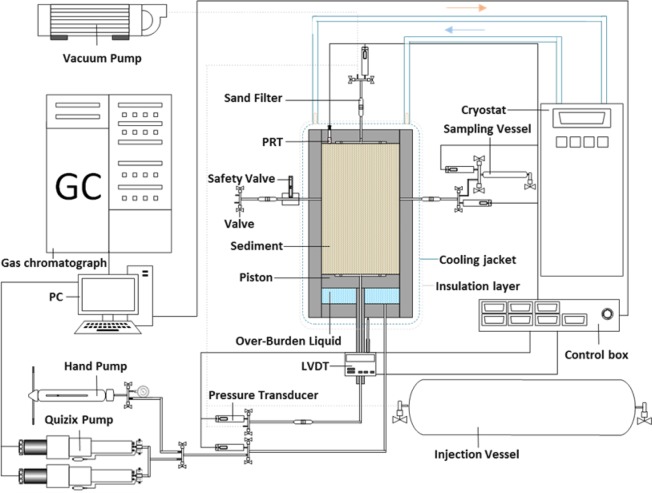
Schematic of the high-pressure cell setup.

A high-pressure cylindrical cell setup was employed in all the experiments as shown in Fig. 5. The cell body is made of 316 stainless steel. The geometric area exposed to the hydrate-bearing sediments has been placed between a fixed top cap and a bottom cap. A movable piston is mounted above the bottom cap to simulate the overburden pressure, moving up and down by withdrawal or injection of hydraulic fluid behind the piston. Thus, the reservoir is not in direct contact with hydraulic fluid. Hydraulic fluid injection/removal was performed using a dual-cylinder Quizix pump (SP-5200, Chandler Engineering) for maintaining the pressure or a hand pump for achieving initial compaction pressure. A linear variable differential transformer (LVDT) is mounted on the bottom cap to measure the piston movement; therefore, the reservoir volume could be calculated at any stage. For controlling the temperature during experiments, the cell is fitted in an aluminium jacket, which is cooled/heated as a whole by circulation of the cooling fluid (water/monoethylene glycol solution from a cryostat (Julabo MA-4). The cooling jacket is wrapped with an insulation layer to reduce the heat transfer from the surrounding environment and the temperature gradient. Two QUARTZDYNE pressure transducers (model QS30K-B, Quartzdyne Inc., U.S.A.)(+/− 0.005 MPa) and a Platinum Resistance Thermometer (PRT) (+/− 0.1 K) were used to measure the cell and overburden pressures, and the cell temperature, respectively. The temperature, all pressures (including pore pressure, overburden pressure, and pump pressure), and the LVDT displacement were monitored by a feedback system of the setup (LabVIEW software from National Instruments). All gas injections, withdrawals, and samplings, were achieved using valves allocated at the top, bottom, and two sides. Finally, a Gas Chromatograph (GC) (Varian 3600, Agilent Technologies) was used to analyse the composition of gas samples for monitoring the evolution in gas composition within the system.

### Procedure

In a typical test, 1076.6 g of the sand was partially saturated with 155.6 g deionized water. The wetted sediment was then loaded into the cell, and the system was evacuated to soak the remained air out. Following evacuation, the piston level was adjusted to compact the system with 3.45 MPa overburden pressure for at least a day. Prior to injection of the test gas, the system temperature was set to 298.15 K (to ensure there was no hydrate formation possibility in the working pressure range). A certain amount of CH_4_ gas was then added through the valves from top and bottom until the desired initial pressure is reached. Subsequently, the system temperature was set to 273.35 K (just above freezing point of water) to form hydrate without appearance of ice in the system. The system pressure reduced as temperature declined. Since water was exposed to the high pressure CH_4_ well inside the HSZ at this point, hydrate formation started, reducing the system pressure till the equilibrium point was reached at the experimental temperature. The onset of hydrate formation appeared at the point where there was a clear change in slope of the pressure profile during cooling. The hydrate formation period could take more than a week because there was no mixing in the system. Although some conventional temperature cycling methods (e.g. Stern’s method^34^) could be used to accelerate the hydrate formation, the cryostat temperature remained stable to preserve the sediment structure built in an entirely controlled method. Hydrate formation/growth continued until a stable pressure profile could be observed, confirming completion of hydrate formation.

After completion of hydrate formation, the first test was conducted at this hydrate formation temperature while for the other experiments, the bath temperature was re-set to the target temperature. At this point, hydrate, gas, water, and ice saturation were calculated as shown in Table 1. The same formulation as our previous work^35^ was used for the calculation of the saturation. For purging the remaining methane gas and reducing the proportion of remaining free methane in the gas phase without dissociation of methane hydrate, flue gas composed of 85.4 mol % N_2_ and 14.6 mol % CO_2_ was injected to the cell at a pressure approximately 10 times the equilibrium pressure of methane hydrate at the target temperature after hydrate formation. Then gas was slowly released from the system until the system was depressurized to 0.7 MPa above flue gas hydrate phase boundary immediately to avoid or minimise formation of flue gas hydrates. Once the methane concentration was less than 15% during the purging process, the injection port was closed and the system pressure was reduced to a specified optimum point by moving the piston backward. These optimum pressures were determined by the method described in the companion paper to this work^10^. At this step, pressure was maintained using a dual-cylinder Quizix pump and samples from the gas phase were collected at pre-determined time intervals to be analysed using a GC. After reaching the equilibrium condition (no change in gas composition for 3 days), the system was depressurized in a stepwise manner to recover the remaining methane. Experiment 2 R was conducted to check the repeatability of the experiments, which followed the same procedure as Experiment 2. However, after reaching equilibrium state at the target pressure, instead of depressurization, the system was heated to room temperature (294.15 K) to investigate dissociation of the formed hydrates.

**Table 1:** Hydrate, gas, water, and Ice + quasi liquid saturation after methane hydrate formation and before flue gas injection.

Experiment No.	1	2	3	4	5	6	7	2 R
Temperature (K)	261.2	264.8	268.6	273.3	278.1	282.1	284.2	264.8
Hydrate saturation (vol%)	67.5	66.8	66.2	60.2	54.4	48.8	47.3	66.9
Gas saturation (vol%)	24.8	25.1	25.6	28.3	26.0	27.1	25.5	25.0
Water saturation (vol%)	0.0	0.0	0.0	11.5	19.6	24.1	27.2	0.0
Ice + Quasi liquid saturation (vol%)	7.7	8.1	8.2	0.0	0.0	0.0	0.0	8.1

**Figure 6:**
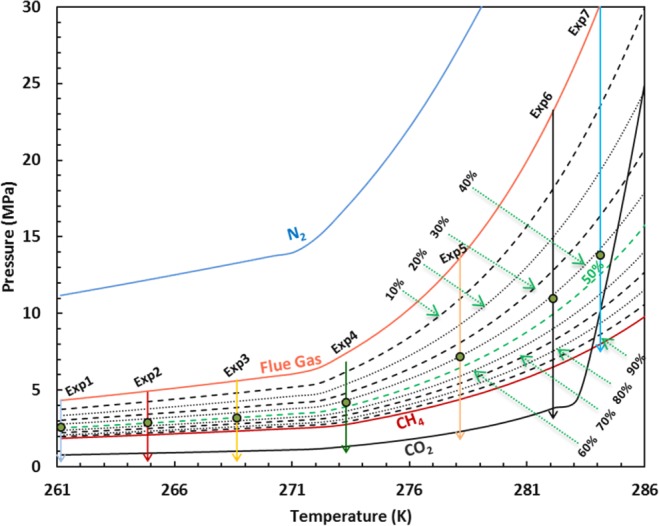
The predicted hydrate stability zones of CO_2_, N_2_, *CH*_4_ and their mixtures and the experimental conditions.

As we have previously defined the effect of pressure on the CO_2_ storage and methane recovery, and have indicated that there is an optimum pressure^10^ at each temperature using simulated bulk conditions, it is necessary to determine the efficiency of the method at real world conditions. Furthermore, it is also important to characterize the kinetics of the process in such conditions as mass and heat transfer is strongly constrained by mesoporous media. I addition, the effect of ice on the kinetics of the process was investigated to cover conditions of encountered in methane hydrate reservoirs located in high-latitude regions of the Earth. Hence, the experiments were designed to fundamentally understand the kinetics and efficiency of methane recovery and CO_2_ storage by injection of flue gas into hydrate-bearing sediments at the previously defined optimum pressures. The procedure was in all cases as described above. The experimental pressure/temperature conditions together with hydrate stability zones of N_2_, CH_4_, CO_2_, flue gas, and different combinations of flue gas/CH_4_ are provided in Fig. 6. The green dots show the optimum conditions at which the system was kept at nearly constant pressure. According to Fig. 6, the experiments sufficiently cover a temperature range for permafrost, sub permafrost, subglacial, and subsea sediments^36^.
